# Intravesical OnabotulinumtoxinA Injection for Overactive Bladder Patients with Frailty, Medical Comorbidities or Prior Lower Urinary Tract Surgery

**DOI:** 10.3390/toxins8040091

**Published:** 2016-03-25

**Authors:** Chun-Hou Liao, Chung-Cheng Wang, Yuan-Hong Jiang

**Affiliations:** 1 Department of Urology, Cardinal Tien Hospital and School of Medicine, Fu-Jen Catholic University, Taipei 24205, Taiwan; 2 Department of Urology, En Chu Kong Hospital, College of Medicine, National Taiwan University, Taipei 23702, Taiwan; ericwcc@ms27.hinet.net; 3 Department of Biomedical Engineering, Chung Yuan Christian University, Chung-Li 32023, Taiwan; 4 Department of Urology, Buddhist Tzu Chi General Hospital and Tzu Chi University, Hualien 97002, Taiwan; redeemer1019@yahoo.com.tw

**Keywords:** botulinum toxin, Type A, urinary bladder, overactive, frail elderly, comorbidity

## Abstract

Overactive bladder (OAB) symptoms increase with age and involve several comorbidities. OnabotulinumtoxinA (BoNT-A) intravesical injection is a treatment choice for patients who are intolerant of or refractory to antimuscarinics. However, the increased risk of urinary tract infection and elevated post-void residual (PVR) volume post-treatment require resolution. Male sex, baseline PVR > 100 mL, and comorbidities are independent risk factors of adverse events (AEs) such as acute urinary retention (AUR). Intravesical BoNT-A injection is safe and effective for OAB patients with frailty, medical comorbidities such as Parkinson’s disease (PD), chronic cerebrovascular accidents (CVA), dementia, or diabetes, or a history of prior lower urinary tract surgery (prostate or transvaginal sling surgery). Post-treatment, 60% of frail elderly patients had a PVR volume > 150 mL and 11% had AUR. Although intravesical BoNT-A injection is safe for PD patients, CVA patients had higher strain voiding rates. Diabetic patients were at increased risk of large PVR urine volume and general weakness post-treatment. Treatment results were similar between patients with and without a history of prostate or transvaginal sling surgery. Possible AEs and bladder management strategies should be conveyed to patients before treatment. Careful patient selection is important, and therapeutic safety and efficacy should be carefully balanced.

## 1. Introduction

Overactive bladder (OAB) syndrome is a symptom complex defined by the International Continence Society as the presence of “urinary urgency, usually accompanied by frequency and nocturia, with or without urgency urinary incontinence (UUI)” [1]. These symptoms are bothersome and negatively affect health-related quality of life (HR-QoL). They may also increase patients’ degrees of anxiety and depression [2]. The prevalence of OAB symptoms increases with age [3]. In elderly patients with OAB symptoms, the risk of falls, fractures, and mortality may also increase [4]. In addition, several medical comorbidities may also be associated with OAB symptoms, including musculoskeletal, neurological, and immunological diseases [5]; congestive heart failure [6]; and diabetes mellitus (DM) [7].

The first-line treatment for OAB is behavioral therapies, while the second-line treatment is oral medication, such as antimuscarinics or β3-adrenoceptor agonists [8,9]. However, patients may notice inadequate improvement or have low treatment compliance to these medication regimens [10]. The intravesical injection of onabotulinumtoxinA (BoNT-A) recently became an effective treatment choice for patients intolerant of or refractory to antimuscarinics [11,12]. However, widespread use of intravesical BoNT-A injection is usually hampered by adverse events (AEs), including elevated post-void residual (PVR) urinary volume and urinary tract infection (UTI) [13]. Patients must be fully informed of the possibility of elevated PVR and acute urinary retention (AUR), particularly those at a higher risk of AEs [14]. Clean intermittent catheterization (CIC) may be initiated. However, poor efficacy and CIC-related issues may contribute to treatment discontinuation [15].

Therefore, identifying and properly managing patients at a high risk of AEs is important to increasing patient satisfaction and preventing complications. Kuo *et al.* reported that male sex, a baseline PVR urinary volume >100 mL, and medical comorbidities are independent risk factors of AEs, including AUR or a large PVR urinary volume [16]. There had been several large randomized controlled studies which provide good evidence and support the use of intravesical BoNT-A in OAB patients [11,12]. This manuscript searches and reviews publications in Pubmed regarding intravesical BoNT-A in specific groups of patients with OAB symptoms, such as frail elderly patients, those with medical comorbidities, and those with a history of prior lower urinary tract surgery. These patients are relatively more susceptible to complications. Most of these selected studies are case-control studies (level of evidence III) or case series (level of evidence IV). All selected reports regarding the safety profiles after intravesical BoNT-A treatment in these patient groups are summarized in Table 1. We hope that this review can provide useful information about the safety and efficacy of intravesical BoNT-A injection in these groups.

## 2. Frail Elderly

The prevalence of OAB symptoms increases with age [3], but treatment in elderly patients is traditionally difficult. Behavioral therapies are often ineffective or difficult to implement. Altered drug solubility, metabolism, and clearance, as well as increased polypharmacy were also noted in elderly patents [27]. The possible risk of AEs related to cognitive function is another important problem of OAB medication in elderly individuals [28]. The cognitive changes induced by antimuscarinics are usually associated with the ability of the drugs to cross the blood–brain barrier and bind to receptors in the central nervous system (CNS). Although cognitive function was not affected by OAB medication in most controlled trials, elderly individuals are often excluded from these studies [29]. An increased risk of dementia was recently reported to be associated with a higher frequency of cumulative antimuscarinics use [30]. For mirabegron, although the risk of CNS-related AEs is not significant, hypertension as a reported AE remains a concern for elderly patients [31].

*Frail elderly* is usually defined as elderly individuals with a clinical presentation or phenotype combining impaired physical abilities, balance, mobility, muscle power, cognition, motor processing, nutrition, and endurance (including feelings of exhaustion and fatigue) [32,33]. OAB treatment is even more difficult in elderly patients with frailty than those without frailty. For frail elderly, Samuelsson *et al.* reported a systemic review of medical treatment for urinary incontinence [34]. Only one drug (oxybutynin) was studied in the frail elderly population with UUI. However, the effect of oxybutynin on urinary incontinence or HR-QoL was not significant. For elderly patients, darifenacin, fesoterodine, solifenacin, tolterodine, and trospium were investigated. Urinary leakage decreased after treatment in these studies. Dry mouth and constipation are the most common AEs. Moreover, they found that no study to date has evaluated the effects of β3-adrenoceptor agonists in elderly patients. Samuelsson *et al.* concluded that antimuscarinics for elderly patients with UUI may have a small but significant effect but that the evidence of the efficacy of these medications for frail elderly patients remains insufficient [34].

To avoid the systemic AEs that accompany oral medication, intravesical BoNT-A injection seems to be an attractive alternative for elderly OAB patients. White *et al.* used intravesical BoNT-A 200 U in elderly patients (18 women and three men with a mean age of 81.2 years) and reported the short-term treatment results [18]. Patients received an intravesical detrusor injection with trigone sparing at 20 sites. The average number of voids per day preoperatively (mean ± SD) was 11.4 ± 1.67, while the average pads used per day preoperatively was 4.0 ± 0.89. Sixteen (76%) of the 21 patients had >50% symptom improvement post-injection for one time 1 month after treatment. In addition, the average daily number of voids (5.19 ± 0.83, *p* < 0.001) and average daily number of pads (1.3 ± 0.60, *p* < 0.001) improved significantly. Of the remaining five patients who did not report significant improvement, a repeat injection resulted in >50% improvement in two. However, the remaining three did not report significant improvement after the second injection. The average duration of symptom deterioration was 7.12 months. There were no serious treatment-related complications. White *et al.* [18] concluded that intravesical BoNT-A is safe and effective for elderly patients.

Liao *et al.* investigated the efficacy and safety of intravesical BoNT-A injections for refractory OAB symptoms among frail elderly, non-frail elderly, and younger patients [21]. In their study, 166 patients received intravesical injections of BoNT-A 100 U. *Frail elderly* was defined as those aged >65 years and who met three or more of the following criteria: unintentional weight loss, self-reported exhaustion, slow walking speed, weakness, and/or low physical activity. The treatment results were similar among younger, non-frail elderly, and frail elderly patients. Significant improvement in UUI and HR-QoL were obtained in frail elderly as well as younger or non-frail elderly patients. However, the risk of large post-treatment PVR urinary volume (>150 mL) was significantly higher in the frail elderly group than in the younger and non-frail elderly groups (60.7% *vs.* 39.7% *vs.* 35.7%, respectively, *p* = 0.018). Urinary retention occurred in seven frail elderly (11.5%), four (6.3%) non-frail elderly, and one younger (2.4%) patient. The recovery time from urinary retention was also significantly longer in the frail elderly patients group. In addition, the cumulative success rate was significantly lower in the frail elderly group than in the other two groups (*p* = 0.009).

Intravesical BoNT-A injection for refractory OAB patients in non-frail elderly and frail elderly groups can be used as an effective treatment option. However, for the frail elderly, there was an increased risk of large post-treatment PVR urinary volume and a lower long-term success rate. CIC may be needed to empty the patient’s bladder if patients wish to be completely dry and avoid the need for an indwelling catheter. The possible AEs and strategy to manage bladder problems should be communicated to frail elderly patients.

## 3. CNS Lesions: Parkinson’s Disease, Chronic Cerebrovascular Accidents, or Dementia

In the elderly, the degeneration of the CNS is considered as a possible pathogenic factor for OAB symptoms [35]. According to a health survey in the community, OAB symptoms were reported in 31% of subjects with CNS lesions. Relatively poorer HR-QoL than general OAB population was found in these OAB patients with CNS lesions [36]. In patients with Parkinson’s disease (PD), lower urinary tract symptoms and bladder dysfunction are the most common autonomic disorders. The estimated incidence of lower urinary tract symptoms is 27%–80% [37]. Both impaired detrusor contractility and sensory perception problems are proposed mechanisms of OAB symptoms in these patients. In addition to OAB symptoms, detrusor hyperreflexia and inadequate contractility or detrusor underactivity (DU) may also be noted in patients with a history of cerebrovascular accident (CVA) or PD. Elevated PVR urinary volume may be noted in these patients [38,39]. These patients may also have concomitant urethral sphincter dyssynergia, poor relaxation of the external urethral sphincter, and poor relaxation of the pelvic floor muscles [40]. These concomitant conditions may also make the use of intravesical BoNT-A more complex in these patients.

Antimuscarinics are the current main OAB medications for patients with CNS lesions as healthy individuals. However, double-blind, placebo-controlled, randomized studies specifically for these patients taking antimuscarinics or β3 agonists are still lacking. The commonly used centrally-acting anticholinergic drugs (e.g., trihexyphenidil) for PD may increase the risk of cognition-associated AEs [41]. Although large amounts of evidence-based data to guide the choice of pharmacological agents for patients with dementia are lacking, dementia was not a proposed barrier to pharmacological treatment, but the total load of antimuscarinics must be considered [42]. Caution was also suggested in the use of antimuscarinics in elderly patients with preexisting dementia [29].

Giannantoni *et al.* [19] evaluated the effects of intravesical BoNT-A for refractory detrusor overactivity (DO) in four patients with PD and two patients with multiple system atrophy (MSA) in 2009. All patients received intradetrusor BoNT-A 200 U injection at 20 sites. All patients had decreased daytime and nighttime urinary frequencies and improved QoL scores 3 months after the injection. However, elevated PVR urinary volume was noted in two patients with MSA that required CIC. These researchers concluded that intradetrusor BoNT-A injection is safe and effective for OAB symptoms induced by PD [19]. In 2011, the same group reported another study using a half dose of intravesical BoNT-A (100 U) injected into eight patients with PD and OAB symptoms. Clinical and urodynamic improvements were noted after treatment, and the results lasted for more than six months [20]. In 2014, Anderson *et al.* [22] treated 20 patients with PD and UUI using BoNT-A 100 U under local anesthesia as an in-office procedure. They concluded that office cystoscopy with BoNT-A 100 U intravesical injection treatment can be considered a potential long-term management strategy for patients with PD and urinary incontinence who are nonresponsive to oral medication [22]. No cases of urinary retention occurred in this study.

Based on previous studies, intravesical BoNT-A injection is considered an effective method to treat intractable OAB symptoms in patients with PD. However, no definite recommendations were made in terms of BoNT-A dosages, AUR risk factors, or voiding difficulties. The long-term effectiveness data are also unavailable. In contrast to the reports conducted in patients with PD, the treatment results for patients with a history of CVA and OAB symptoms are relatively insufficient. For patients with a history of CVA and OAB symptoms, one previous report showed that complete continence after a BoNT-A 200 U intravesical injection was obtained in only 8.3% of patients. Only 50% of patients experienced urodynamic improvement [17]. In 2014, Jiang *et al.* [24] reported the safety and efficacy of intravesical BoNT-A injection in elderly patients with chronic CNS lesions and OAB symptoms. In this retrospective analysis, 40 patients with OAB symptoms due to CVA (*n* = 23), PD (*n* = 9), and dementia (*n* = 8) were enrolled, while 160 age-matched patients without chronic CNS lesions were used as a control group. The improvements in urgency severity scale score, increases in bladder capacity, and increases in PVR urinary volume were similar among these groups three months post-treatment. The risk of AUR or UTI after treatment did not increase in patients with chronic CNS lesions. However, a higher risk of straining to void became more common in patients with a history of CVA and OAB symptoms. There was no significant difference in long-term success rates between the patients with chronic CNS lesions and those without chronic CNS lesions [39].

The overall rates of AEs related to intravesical BoNT-A injection for patients with chronic CNS lesions and OAB symptoms seem acceptable. In addition, the long-term effects were also similar between those with and without chronic CNS lesions. Nonetheless, before intravesical BoNT-A treatment is chosen for this very vulnerable population, the possibility of longstanding urinary retention and chronic catheterization requires especially careful evaluation.

## 4. Diabetes Mellitus

DM was considered an independent risk factor of OAB symptoms in an epidemiological study [43]. More persistent and adherent to OAB medication were noted in diabetic patients. Diabetic patients also had a 16.6% higher odd of receiving a second course of prescription for OAB symptoms [44]. In addition, more bothersome OAB symptoms may be noted in diabetic patients, who may require a greater amount of medical help. Nonetheless, conventional treatment, including oral medication, is usually not as effective for diabetic patients with OAB symptoms as for normal OAB patients.

Intravesical BoNT-A injection can be considered a treatment choice for diabetic patients with DO that is refractory to oral medication. However, it remains unknown whether diabetic patients are more or less sensitive to intravesical BoNT-A injection. Wang *et al.* reported an age-matched case control study that investigated the safety and efficacy of intravesical injection BoNT-A in patients with DM and refractory OAB symptoms [23]. A total of 48 patients with type 2 DM and another 48 age-matched controls received BoNT-A 100 U. Similar successful results were noted at six months post-treatment (DM 56% *vs.* non-DM 61%, *p* = 0.128). However, there was a significantly higher risk of large PVR urinary volumes (DM 60.4% *vs.* non-DM 33.3%; *p* = 0.007) and general weakness (DM 10.4% *vs.* non-DM 0%; *p* = 0.03) after treatment in diabetic patients. In addition, the baseline urodynamic parameters could not be used as predictors for the occurrence of AEs in diabetic patients. No serious complications were noted in either group.

Intravesical BoNT-A injection seems to be an effective and safe treatment for patients with DM and OAB symptoms that are refractory to oral medication. Although DM itself did not influence the treatment outcomes or overall AE rates, the incidences of general weakness and large PVR urinary volume post-treatment were increased in diabetic patients. Patients with DM should be informed of the possible increased risk of large PVR urinary volume before undergoing intravesical BoNT-A injection.

## 5. Prior Prostate Surgery

While the efficacy and safety of intravesical BoNT-A for patients with OAB that is refractory to oral medication have been investigated in several studies, the efficacy and safety data in the male population is relatively insufficient. Only female patients were enrolled in many studies, and a female dominance was noted in studies with mixed-sex populations [45]. Due to the differences in anatomy and urological pathology between men and women, different treatment results may be observed between them. Kuo *et al*. had reported that male sex is an independent risk factor of higher risk of large PVR urinary volume or AUR post-treatment [16]. In addition, significant differences were observed within the male population that included those with a history of prior radiotherapy for prostate cancer and those with a history of prior prostate surgery (radical prostatectomy, RP, or transurethral resection of prostate, TURP).

Habashy *et al.* reported intravesical BoNT-A injection for 43 men with non-neurogenic OAB, including 20 (47%) who had prior prostate surgery, of whom 11 had RP and nine had TURP [25]. Men with a history of prior prostate surgery had similar Patients’ Global Impression of Improvement (PGI-I) scores to those without prior prostate surgery (2.6 ± 0.5 *vs.* 2.8 ± 0.5, respectively, *p* = 0.6). Men with previous TURP had significantly higher PGI-I scores post-treatment than those with previous RP (3.3 ± 0.8 *vs.* 2.0 ± 0.5, respectively, *p* < 0.05). In addition, only patients with a history of prior RP had a significant reduction in daily pad use (from 3.5 ± 1.7 to 1.6 ± 0.9 pads/day, *p* < 0.05), while patients with a history of prior TURP did not have a significant reduction in daily pad use (from 1.7 ± 1.5 to 1.4 ± 1.5 pads/day, *p* = 0.4). The authors concluded that intravesical BoNT-A injection in men with OAB symptoms that are refractory to oral medication can achieve significant symptom improvement despite a history of prior prostate surgery. Nonetheless, those with a history of prior RP benefited more than those with a history of prior TURP in both daily pad use and PGI-I score [25]. Considering AEs after intravesical BoNT-A injection, Kuo *et al*. reported that the risk of specific AEs, such as AUR, large PVR, hematuria, UTI, or general weakness was similar between men with and those without a history of prior TURP [16]. Thus, OAB patients with a history of prior prostate surgery seem suitable for intravesical BoNT-A injection.

## 6. Prior Transvaginal Sling Surgery

Transvaginal mid-urethral slings (MUS) recently became generally accepted as a preferred surgical treatment option for women with stress urinary incontinence (SUI) [46]. Although the overall complication rate was low for these procedures, *de novo* OAB symptoms were found in 6%–8% of women receiving MUS for SUI [47]. The pathophysiology of *de novo* OAB symptoms after MUS surgery has yet to be elucidated. The symptoms may occur even in patients undergoing their first MUS surgery in whom the tape is correctly placed [48]. Recent evidence also suggests that patients with *de novo* OAB symptoms reported less subjective benefit from antimuscarinics treatment than those with idiopathic OAB symptoms [49].

Miotla *et al.* [26] compared the efficacy of BoNT-A between women with *de novo* OAB symptoms after MUS surgery and women with idiopathic OAB symptoms. All patients received intravesical BoNT-A 100 U injection at 20 sites. A total of 22 patients (41.5%) of the idiopathic OAB group and 19 patients (38.8%) of the *de novo* OAB groups were completely dry 12 weeks post-treatment. Significant decreases in the average daily number of voids (−2.39 *vs.* −2.0) and daily incontinence episodes (−1.38 *vs.* −1.44) were achieved in both groups (*p* < 0.001). However, there was no significant difference between these two groups. An increased average voided volume of > 90 mL was observed in both groups. Four patients developed urinary retention: three with *de novo* OAB symptoms and one with OAB symptoms. The risk of UTI and general weakness were also low in both groups.

Intravesical BoNT-A injection can result in similar improvement of OAB symptoms in patients with *de novo* OAB symptoms as well as those with idiopathic OAB symptoms. The risk of urinary retention and requirement for CIC were acceptable even for women with a prior MUS surgery and *de novo* OAB symptoms. Intravesical BoNT-A 100 U injection can be a treatment choice for patients with *de novo* OAB symptoms after MUS surgery. Some patients have mixed SUI and UUI, their storage symptoms may persist or even worsen after MUS. The use of intravesical BoNT-A may have some potential benefits for these patients, although there is still no clinical report at present.

## 7. Conclusions

In general, intravesical BoNT-A injection for OAB patients with frailty, medical comorbidities (PD, chronic CVA, dementia, or DM), or a history of prior lower urinary tract surgery (prostate or transvaginal sling surgery) is safe and effective. However, the risk of large PVR urinary volume after treatment increased and the long-term success rate was relatively lower in frail elderly patients. In addition, although intravesical BoNT-A injection for PD patients is safe, patients with a history of CVA and OAB symptoms had a higher incidence of straining to void. The incidence of a large PVR urinary volume and general weakness after treatment were also increased in the patients with DM. In contrast, the treatment results were similar between the patients with and those without a history of prior prostate or transvaginal sling surgery. All of these possible AEs and strategies to manage bladder problems after treatment should be communicated to patients before treatment initiation. Careful patient selection is important, and the balance between therapeutic safety and efficacy should be maintained.

## Figures and Tables

**Table 1:** Summary of efficacy and safety profiles after intravesical onabotulinumtoxinA (BoNT-A) treatment in specific patient groups.

Author (Year)	Patient (n)	Dose	Global Response or Dry Rate	AUR	Large PVR	Strain to Void	Hematuria	UTI	Weakness
Kuo (2006) [17]	CVA (12)	200 U	3 months Dry 8.3%	-	-	-	-	-	-
Kuo (2009) [16]	Comorbidity	100–200 U	6 months Success 61%	12%	* 61%	52%	10%	13%	7%
	No comorbidity		70%	6%	* 39%	43%	7%	16%	0%
	TURP		6 months Success 74%	14%	51%	51%	* 4%	* 2%	0%
	No TURP		60%	18%	47%	49%	* 16%	* 13%	2%
White (2008) [18]	Elderly (21)	200 U	1 month Success 76%	0%	-	-	-	9.5%	-
Giannantoni (2009) [19]	PD (2)	200 U	-	0%	-	50%	-	0%	0%
	MSA (4)			50%	-	-	-	0%	0%
Giannantoni (2011) [20]	PD (8)	100 U	-	25%	-	-	-	-	-
Liao (2013) [21]	Frail elderly (61)	100 U	12 months Success * 6.8%	11.5%	* 60.7%	45.9%	13.1%	13.1%	* 6.6%
	Elderly (63)		22.3%	6.3%	39.7%	41.3%	11.1%	9.5%	0.0%
	Young (42)		23.1%	2.4%	35.7%	23.8%	4.8%	28.6%	0.0%
Anderson (2014) [22]	PD (20)	100 U	3 months Success 59%	0%	15%	-	-	10%	0%
Wang (2014) [23]	DM (48)	100 U	6 months Success 56%	10.4%	* 60.4%	54.2%	8.3%	12.5%	* 10.4%
	Non-DM (48)		61%	6.3%	* 33.3%	41.7%	10.4%	12.5%	* 0%
Jiang (2014) [24]	Control (160)	100 U	-	10%	39.3%	50.6%	10.0%	13.8%	3.8%
	CNS lesion (40)			-	-	-	-	-	-
	CVA (23)			17.4%	52.2%	* 73.9%	8.7%	4.3%	4.3%
	PD (9)			11.1%	33.3%	11.3%	11.1%	22.2%	11.1%
	Dementia (8)			0.0%	12.5%	25.0%	0.0%	0.0%	0.0%
Habashy (2015) [25]	Male NNOAB (43)	100–300 U	PGI-I	-	-	-	-	-	-
	No Surgery (23)		2.8 ± 0.5						
	Surgery (20)		2.6 ± 0.5						
Miotla (2015) [26]	Prior MUS (49)	100 U	3 months Dry 38.8%	4.0%	26.5%	-	6.1%	4.0%	2.0%
	Idiopathic OAB (53)		41.6%	0.0%	15.1%		1.8%	3.7%	0.0%

* *p* < 0.05 compared between groups in the same study; CVA: cerebral vascular accident; TURP: transurethral resection of prostate; PD: Parkinson’s disease; MSA: multiple system atrophy; DM: diabetes mellitus; CNS: central nervous system; NNOAB: non-neurogenic overactive bladder; MUS: midurethral sling; OAB: overactive bladder; AUR: acute urinary retention; PVR: postvoid residual; UTI: urinary tract infection; PGI-I: Patient Global Impression of Improvement score.
